# Supplementary material for: Antioxidant Efficacy of a Spirulina Liquid Extract on Oxidative Stress Status and Metabolic Disturbances in Subjects with Metabolic Syndrome
Source: Mar Drugs. 2022 Jul 1;20(7):441. doi: 10.3390/md20070441 (PMC9318250; doi:10.3390/md20070441)
Supplement: Supplementary file 1 [file marinedrugs-20-00441-s001.zip › marinedrugs-1724184-supplementary.pdf]

Table S1. Composition Group

Randomization number	Product	BMI (kg/m²)	Waist circumference (cm)	Triglycerides (g/L)	HDL cholesterol (g/L)	Glycemia (mmol/L)
**2**	Placebo	30,2447777	95	2,74	0,45	5,39
**4**	Placebo	31,6608997	100	2,59	0,42	5,445
**5**	Placebo	27,1368889	83	1,7	0,56	4,675
**8**	Placebo	27,0703125	90	1,75	0,72	5,335
**10**	Placebo	25,9121323	96	2,07	0,34	5,005
**15**	Placebo	26,1285041	101	0,98	0,51	5,225
**16**	Placebo	31,8827161	101	1,09	0,49	5,665
**14**	Placebo	34,1985568	114	2,6	0,38	4,565
**17**	Placebo	32,0298092	101	3,12	0,49	4,84
**18**	Placebo	24,9787415	87	2,29	0,48	4,015
**22**	Placebo	31,3779892	100	0,8	0,36	4,18
**24**	Placebo	31,5711195	107	0,8	0,85	5,06
**26**	Placebo	29,0021633	102	0,89	0,46	5,005
**27**	Placebo	34,2976067	106	0,89	0,44	5,335
**29**	Placebo	32,1108238	102	2,05	0,4	5,39
**30**	Placebo	30,9617288	108	2,55	0,48	6,325
**33**	Placebo	32,158538	103	1,28	0,46	5,61
**35**	Placebo	27,3114669	100	1,48	0,45	5,72
**39**	Placebo	26,9387755	95	1,46	0,37	5,885
**38**	Placebo	29,1591837	101	3,45	0,27	6,6
**1**	Spirulysat®	27,8469997	90	0,96	0,75	5,5
**3**	Spirulysat®	34,9387755	104	1,15	0,57	5,445
**7**	Spirulysat®	26,2037037	99	1,63	0,49	5,72
**6**	Spirulysat®	33,3279923	101	1,64	0,52	6,38
**9**	Spirulysat®	32,5026673	94	1,14	0,37	6,05
**13**	Spirulysat®	28,5705565	100	0,94	0,44	4,51
**11**	Spirulysat®	25,0137741	89	0,88	0,57	5,555
**12**	Spirulysat®	27,1642888	96	2,04	0,47	5,115
**21**	Spirulysat®	27,993022	95	1,54	0,56	5,335
**19**	Spirulysat®	29,4415901	105	0,45	0,59	5,445
**25**	Spirulysat®	27,9320793	101	1,59	0,58	6,215
**20**	Spirulysat®	27,3008459	100	1,11	0,47	4,235
**23**	Spirulysat®	29,1035695	101	2,35	0,59	5,995
**28**	Spirulysat®	27,8604468	95	1,68	0,71	5,005
**31**	Spirulysat®	33,4883431	109	1,1	0,58	5,5
**32**	Spirulysat®	31,9559229	101	1,37	0,53	5,94
**34**	Spirulysat®	31,1613894	111	1,04	0,54	6,215
**37**	Spirulysat®	28,7642046	102	1,12	0,52	
**36**	Spirulysat®	32,5963719	106	1,23	0,46	5,5
**40**	Spirulysat®	29,8211416	99	2,08	0,51	5,94
